# Diffuse type testicular seminoma in a stallion

**DOI:** 10.1007/s00580-016-2316-z

**Published:** 2016-08-09

**Authors:** G. Farjanikish, M. Sayari, A. Raisi, S. Shirian

**Affiliations:** 1 Department of Pathobiology, Faculty of Veterinary Medicine, Lorestan University, Khorram Abad, Iran; 2 Department of Pathology, School of Veterinary Medicine, Shiraz University, Shiraz, Iran; 3 Department of Clinical Studies, Faculty of Veterinary Medicine, Lorestan University, Khorram Abad, Iran; 4 Department of Pathobiology, Faculty of Veterinary Medicine, Shahrekord University, Shahrekord, Iran; 5 Shiraz Molecular Pathology Research Centre, Dr. Daneshbod Laboratory, Shiraz, Iran

**Keywords:** Stallion, Seminoma, Histopathology, Immunohistochemistry

## Abstract

A 7-year-old stallion with progressive left testicular enlargement was presented. Grossly, the excised testicle measured 25 × 15 × 12 cm and weighed 3.7 kg. It was multinodular with a gray-white surface; however, the right testis was normal. Histologically, the neoplastic cells were disseminated diffusely in the tumoral stroma with a minimal fibrovascular stroma. Neoplastic cells were round to polygonal with abundant eosinophilic cytoplasm and large round to oval vesicular or hyperchromatic nucleous with a single prominent nucleolus. Immunohistochemically, the neoplastic cells were positive nuclear immunostaining for C-KIT and negative for OCT3/4. According to gross, histopathological, and immunohistochemical characteristics, the diffuse type of seminoma was diagnosed. Nine months later, the follow-up observation of the case showed that the tumor had no recurrence and metastasis.

## Introduction

Testicular tumors arise from germ cells and sex-cord stromal elements of the testis (MacLachlan and Kennedy 2002) and are divided into four general categories: germ cell tumors, sex-cord stromal tumors, mixed germ cell sex-cord stromal tumors, and primary tumors not specific to the testis (Peters et al. 2001; Rosai 2004). Seminomas arise from the germ cells of the testicular spermatic epithelium mostly occurring in adult or older animals’ retained testicles and are considered benign. They also may be single, multiple, unilateral, bilateral, or cystic (Erer et al. 1992; O’Keefe 1997; Beck et al. 2001). Regarding their histological appearance, these tumors are subdivided into intratubular and diffuse types (MacLachlan and Kennedy 2002). Seminomas are indicative of a low metastatic character (6 % of cases) and rarely trigger paraneoplastic syndrome manifested by alopecia, hyperpigmentation, prostatic squamous metaplasia, diabetes mellitus (Johnston et al. 2001), and bone marrow aplasia because of the presence of hyperestrogenism (Nelson and Couto 1998). Clinical symptoms of the seminoma include abdominal and local pain, anorexia, lethargy, vomiting, dysuria, marching dysfunction, and hyperthermia (Macintire et al. 2005).

In this paper, morphopathological and immunohistochemical characteristics of diffuse form of seminoma in a stallion are described.

## Materials and methods

A 7-year-old stallion with progressive left testicular enlargement was presented. According to the owner, the stallion’s testicle became larger than the normal size over the last 3 months. Considering the physical examinations, the stallion was emaciated and his respiration, heart rate, urination, and reflexes were in a normal range. According to a complete blood count (CBC) test, the percentage of lymphocytes, monocytes, neutrophils, and eosinophils were 37, 1, 63, and 2 %, respectively. Further, the WBC, PCV, and TP were 6890/μl, 31 % and 6 g/dL, respectively. Both testes were descended and the right testis was grossly normal. After physical examination, the horse was presented to the department of surgery for medical castration. Following premedication with acepromazine (0.1 mg/kg, IV) and xylazine (1.1 mg/kg, IV), the mixture of ketamine (2.2 mg/kg, IV) and xylazine (0.5 mg/kg, IV) was used for general anesthesia. The horse was positioned in lateral recumbency with its upper rear limb pulled forward and secured with a rope. Aseptic preparation of the entire scrotal area was routinely performed; the scrotum was anesthetized by subcutaneous direct infiltration along the lines of proposed incision, using 8 ml of a 2 % lidocaine solution into spermatic cords. Testes were compressed against the bottom of the scrotum and two parallel 10-cm-long incisions were placed 2 cm on either side of the raphe along the line of local anesthetic from cranial to caudal poles of the testes. Incising of tunica vaginalis, the ligament of the tail of the epididymis, mesorchium, and mesofuniculum were bluntly transected. Then, the exteriorization of testis, epididymis, and distal portion of the spermatic cord was completed. Two transfixing ligatures were placed 1 cm apart, as far proximally as possible, on the testicular vasculature and ductus deferens by a two vicryl suture. The spermatic cord was crushed for 5 min to achieve hemostasis and then removed by using a Reimer emasculator (crushing component was proximal to the cutting blade). Finally, healing by secondary intention, the scrotal incisions were left unsutured. The horse recovered safely from general anesthesia. After surgery, for the first 24 h after castration, the horse was restricted to prevent hemorrhage from the severed testicular and scrotal vessels. For the postoperative medication, penicillin G procaine and phenylbutazone (2 mg/kg, IV) were administered for 5 and 3 days, respectively. The obtained mass was sent to the department of pathology for further study. Tissue specimen was fixed in 10 % neutral buffered formalin, routinely processed, embedded in paraffin, sectioned at 5 μm, stained by hematoxylin and eosin, and examined by light microscope.

According to the labeled polymer technique, immunohistochemical staining was performed on representative tissue block containing tumor by using Novolink with DAB as chromogen. For immunohistochemical analyses, monoclonal mouse anti-human OCT3/4 and polyclonal rabbit anti-human c-KIT antibodies were used. All antibodies were produced by DAKO (Glostrup, Denmark). Sections (4-um thick) were used for the IHC analysis. The slides were deparaffinized in xylol and then rehydrated and treated by 3 % hydrogen peroxide solution for 10 min at room temperature to quench the endogenous peroxides. The microwave pretreatment antigen retrieval (power 100 for 10 min, then, power 20 for 20 min) was conducted by using a 10-mmol/L concentration of citrate buffer (pH .0). The primary antibody was injected for 1 h (diluted 1:200). The detection system Envision+ (DakoCytomation, Glostrup, Denmark) was used and developed with diaminobenzidine (DakoCytomation). Diaminobenzidine–hydrogen peroxide was applied as the chromogen. The slides were then counterstained by Mayer’s hematoxylin. Subsequently, they were dehydrated and coverslipped.

## Results

**Fig. 1:**
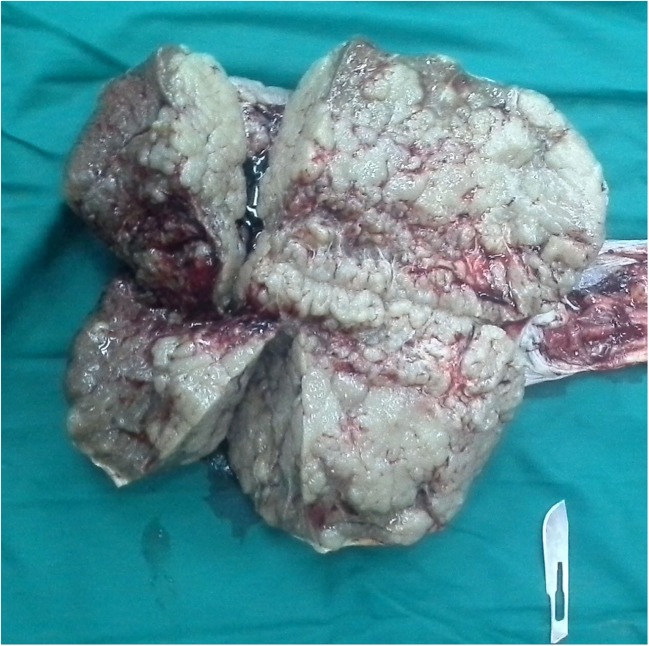
The cross-sections of the neoplastic testicle show the multilobular grayish white areas in various sizes with well-defined borders

In gross examination, the left testis was enlarged about 3–4 times the right one. The excised testicle measured 25 × 15 × 12 cm and weighed 3.7 kg. It was multinodular with a gray-white surface; however, the right testis was normal. The neoplastic testicle cross-sections representing the multilobular grayish white areas were of different sizes with well-defined borders (Fig. 1).

**Fig. 2:**
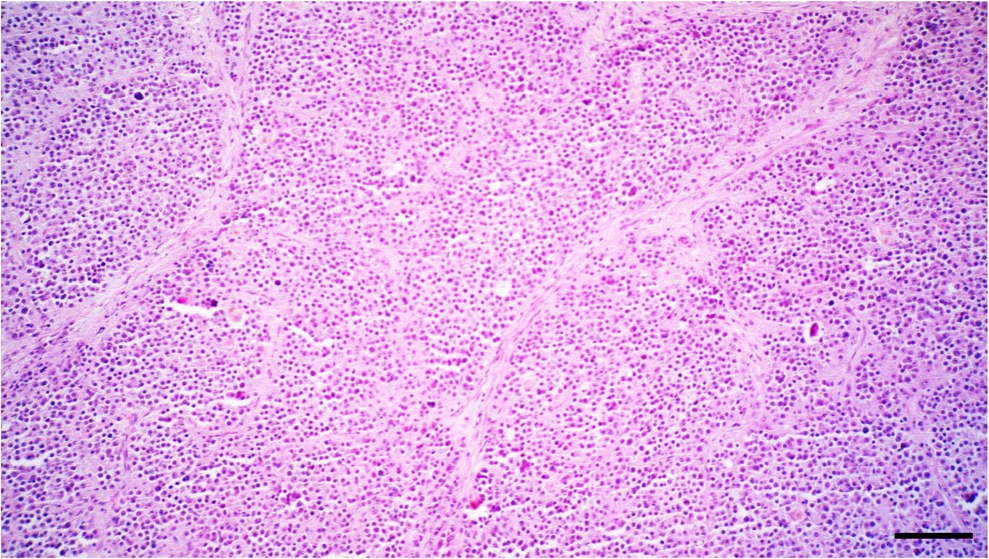
The neoplastic cells are disseminated diffusely in the tumoral stroma with a fibrovascular stroma. HE. Bar, 150 μm

**Fig. 3:**
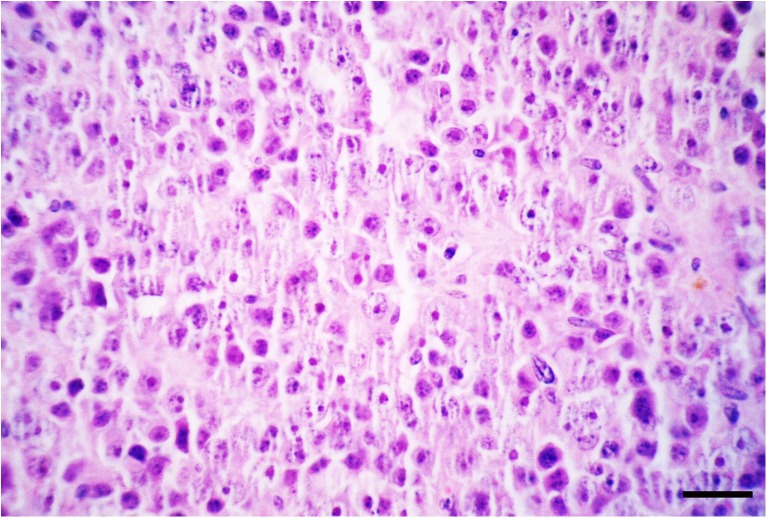
Neoplastic cells are round to polygonal with abundant eosinophilic cytoplasm and large round to oval vesicular or hyperchromatic nucleous with a single prominent nucleolus. HE. Bar, 40 μm

**Fig. 4:**
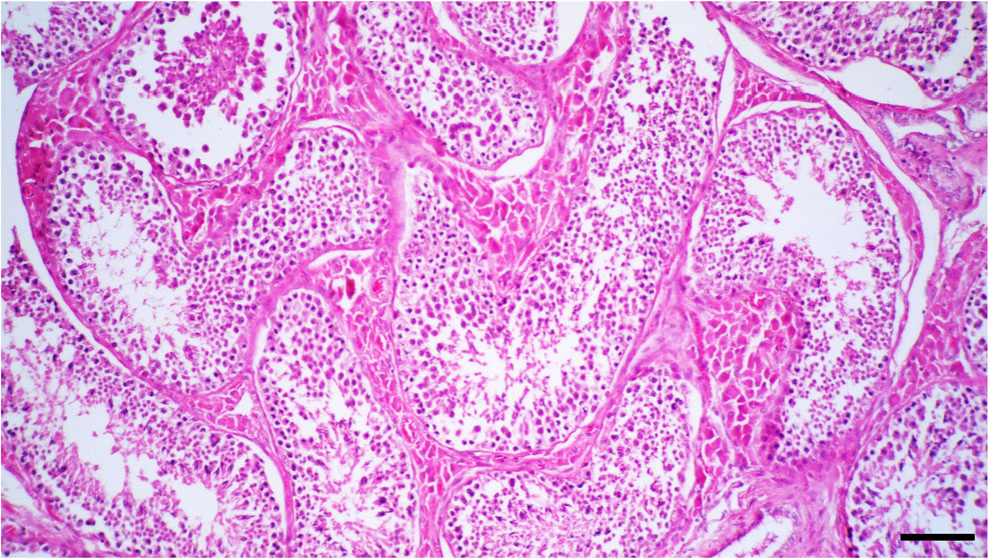
The normal right testis. HE. Bar, 125 μm

**Fig. 5:**
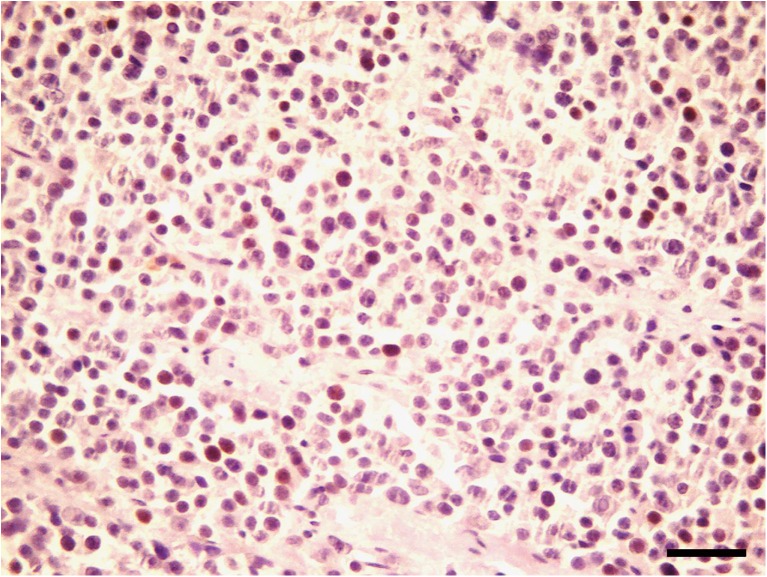
The neoplastic cells show nuclear immunostain for C-KIT. IHC. Bar, 50 μm

Histologically, the neoplastic cells were disseminated diffusely in the tumoral stroma with a fibrovascular stroma (Fig. 2). Neoplastic cells were round to polygonal with abundant eosinophilic cytoplasm and large round to oval vesicular or hyperchromatic nucleous with a single prominent nucleolus (Fig. 3). Mitotic figures were generally rare; also, anisocytosis and anisokaryosis were remarkable. In some areas, multiple areas of necrosis were scattered throughout the tumor, accompanied by moderate infiltrations of plasma cells and lymphocytes. No gross or histopathological lesion was observed in the right testis (Fig. 4). Immunohistochemically, the neoplastic cells were positive nuclear immunostaining for C-KIT (Fig. 5) but were negative for OCT3/4.

Nine months later, the follow-up observation of the case showed that the tumor had no recurrence and metastasis.

## Discussion

The most common causes of scrotal enlargement among animals include hernia scrotalis, funiculus spermaticus torsion, spermatocele, hydrocele, orchitis, and testis/testes tumors (Hollet 2006).

Seminomas have been previously reported in dogs and cats and rarely in rams, horses, and other domestic animals (Anderson et al. 1990; Beck et al. 2001; MacLachlan and Kennedy 2002). Seminoma is a unilateral, single, often benign tumor of the testis; however, malignant forms of the tumor have been reported in rare cases. In this case, gross and histopathological examinations revealed the formation of the tumor in left testis.

Seminomas arise from the germ cells constituting the spermatogenic epithelium within the seminiferous tubules, and, according to the WHO classification and with regard to their histological appearance, they are subdivided into intratubular and diffuse types (MacLachlan and Kennedy 2002; Maiolino et al. 2004; Grieco et al. 2007). Considering the histopathological patterns, the tumor in the present case was diagnosed as a diffuse-type seminoma. The macroscopic and microscopic findings of the seminoma were similar to the results of previous studies (Erer et al. 1992; Beck et al. 2001).

Seminomas are characterized by the presence of fibrous septa-rich in lymphocytes, plasma cells, or occasional granuloma-layering tumor cells sheets. Only rarely tubular structures are encountered. The tumor cells are large and uniform with abundant clear cytoplasm, large centrally located nucleus, and inconspicuous nucleolus.

Seminomas are classified as benign or malignant with regard to their pleomorphic changes, mitotic activity, and metastatic characteristics (Grieco et al. 2007). In the current study, the pleomorphic changes were identified with moderate mitotic activity (Grieco et al. 2007). In mammals, metastasis often occurs to the regional lymph nodes; however, widespread dissemination can also occur to internal organs (MacLachlan and Kennedy 2002). In the present case, no finding was reported on malignancy in pre-operation and 9 months postoperation examinations. For the malignant seminoma cases with metastasis, radiotherapy and chemotherapy are recommended and castration is preferred for the cases without metastasis (Morrison 2002).

In the present study, immunolabeling showed that the neoplastic cells were immunoreactive for C-KIT, as reported in human cases (Woodward et al. 2004; Emerson and Ulbright 2005; Nakai et al. 2005; Lau et al. 2007). C-KIT immunoreactivity of seminoma has been reported to be positive in dogs and cats (Miller et al. 2007; Thorvaldsen et al. 2012; Hohsteter et al. 2014)**.** C-KIT is a product of the c-KIT oncogene, which encodes a type III transmembrane tyrosine kinase receptor that is required in normal spermatogenesis. High expression of c-KIT is found in human seminoma. Our result demonstrates that c-KIT is potently expressed and is potentially a specific tumor marker in equine seminoma, similar to human tumors.

Although OCT3/4 is regarded as the most suitable immunohistochemical markers in human germ cell tumors including seminoma (Cheng 2004; Sung et al. 2006), these were either not expressed in the equine seminoma presently examined. Similar to the present study, immunoreactivity for OCT has also been described to be negative in canine seminoms (Yu et al. 2009). One interpretation of these results might be that all the antibodies used in this experiment were produced against human proteins. As a result, these antibodies might fail to detect canine OCT3/4.
